# Poly(dithiophosphate)s, a New Class of Phosphorus- and Sulfur-Containing Functional Polymers by a Catalyst-Free Facile Reaction between Diols and Phosphorus Pentasulfide

**DOI:** 10.3390/ijms232415963

**Published:** 2022-12-15

**Authors:** Ákos Szabó, Györgyi Szarka, László Trif, Benjámin Gyarmati, Laura Bereczki, Béla Iván, Ervin Kovács

**Affiliations:** 1 Polymer Chemistry and Physics Research Group, Institute of Materials and Environmental Chemistry, Research Centre for Natural Sciences, Magyar tudósok krt. 2, H-1117 Budapest, Hungary; 2 Functional Nanoparticles Research Group, Institute of Materials and Environmental Chemistry, Research Centre for Natural Sciences, Magyar tudósok krt. 2, H-1117 Budapest, Hungary; 3 Soft Matters Group, Department of Physical Chemistry and Materials Science, Faculty of Chemical Technology and Biotechnology, Budapest University of Technology and Economics, Műegyetem rkp. 3, H-1111 Budapest, Hungary; 4 Plasma Chemistry Research Group, Institute of Materials and Environmental Chemistry, Research Centre for Natural Sciences, Magyar tudósok krt. 2, H-1117 Budapest, Hungary; 5 Chemical Crystallography Research Laboratory, Research Centre for Natural Sciences, Magyar tudósok krt. 2, H-1117 Budapest, Hungary

**Keywords:** poly(dithiophosphate), phosphorus pentasulfide, diols, THF incorporation, polythiophosphate, phosphorus-containing polymer, sulfur-containing polymer, catalyst-free, polycondensation, viscoelastic polymer, tunable properties

## Abstract

Novel poly(dithiophosphate)s (PDTPs) were successfully synthesized under mild conditions without any additive in the presence of THF or toluene diluents at 60 °C by a direct, catalyst-free reaction between the abundant phosphorus pentasulfide (P_4_S_10_) and glycols such as ethylene glycol (EG), 1,6-hexanediol (HD) and poly(ethylene glycol) (PEG). GPC, FTIR, ^1^H and ^31^P NMR analyses proved the formation of macromolecules with dithiophosphate coupling groups having P=S and P-SH pendant functionalities. Surprisingly, the ring-opening of THF by the P-SH group and its pendant incorporation as a branching point occur during polymerization. This process is absent with toluene, providing conditions to obtain linear chains. ^31^P NMR measurements indicate long-time partial hydrolysis and esterification, resulting in the formation of a thiophosphoric acid moiety and branching points. Copolymerization, i.e., using mixtures of EG or HD with PEG, results in polymers with broadly varying viscoelastic properties. TGA shows the lower thermal stability of PDTPs than that of PEG due to the relatively low thermal stability of the P-O-C moieties. The low *T*_g_s of these polymers, from −4 to −50 °C, and a lack of PEG crystallites were found by DSC. This polymerization process and the resulting novel PDTPs enable various new routes for polymer synthesis and application possibilities.

## 1. Introduction

**Scheme 1:**
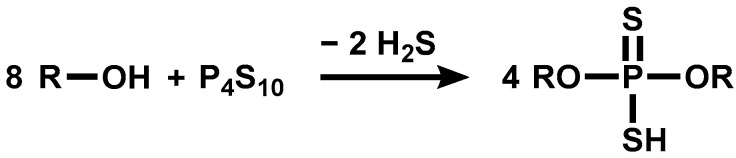
The reaction of phosphorus pentasulfide (P_4_S_10_) with an alcohol.

Macromolecules containing phosphorus and/or sulfur, either in the main chain or pendant, have been intensively investigated worldwide in recent years. This is mainly due to their broad application possibilities, ranging from flame retardancy and environment protection to various high-value-added specialty fields, such as biomaterials, drug delivery, sensors, optical, battery and other energy-related areas, etc. (see, e.g., [1,2,3,4,5,6,7,8,9,10,11,12,13,14,15,16,17,18,19] and the references therein). Although the reaction of phosphorus pentasulfide (P_4_S_10_), a widely available commercial material, with alcohols has been known for a long time [20] to result in the formation of dialkyl dithiophosphates (DTPs), i.e., O,O-diesters of dithiophosphoric acid [21] (Scheme 1), surprisingly, systematic investigations on the utilization of this process for polymer synthesis have not been reported yet, according to the best of our knowledge. Low molecular weight compounds of this kind, especially zinc dialkyl dithiophosphate salts [22,23,24], were recently applied to the preparation of tribological additives as lubricants. Furthermore, the resulting dialkyl dithiophosphates can also be used for several other purposes, e.g., flotation additive [25], wastewater treatment [26] or reagent in organic reactions [27]. The DTPs possess a strong acidic group, and moreover, their anions are highly efficient metal ion complexing agents due to the two sulfur atoms bound to the phosphorus atom [28]. Thus, it can be presumed that the versatility of these functional groups may enable broad application possibilities, especially when they are involved in macromolecular structures.

The synthesis of polymers with dialkyl dithiophosphate moieties can be achieved by different strategies. One of the possibilities is the coupling of dialkyl dithiophosphate complexes of metal ions by using bridging ligands, e.g., 4,4′-bipyridine [29]. The other strategy is the reaction of P_4_S_10_ with diols. This is analogous to the coupling of poly(ethyleneimine) chains by using P_4_S_10_ [9]. However, a multibranched non-acidic polymer was formed with thiophosphoric acid triamide groups in this case. Surprisingly, only a single publication can be found in the literature on poly(dithiophosphate)s. The reactions of diethyldithiophosphate and P_4_S_10_ with diols in bulk at high temperatures (110–140 and 120–180 °C, respectively) were studied by Pudovik et al. [30], who reported on the formation of cyclic dithiophosphate diester products. These authors also claimed the formation of macromolecules, i.e., poly(dithiophosphate)s (PDTPs), but without detailed structural investigations. However, most likely because of the applied high temperatures, this simple method, that is, using the abundant reagents, i.e., diols and P_4_S_10_, has not become a known polymerization technique, in contrast to the reaction of phosphoric acid with diols, which were thoroughly investigated by Penczek and his coworkers [31,32].

Herein, we present the synthesis and characterization of polymers obtained by the reaction of P_4_S_10_ and various α,ω-diols, such as poly(ethylene glycol) with *M*_n_ = 400 g/mol (PEG400), ethylene glycol (EG) and 1,6-hexanediol (HDO) under mild conditions. The property tunability by using different diols simultaneously in the polymerization process, i.e., by copolymerization, is also explored. These systematic investigations demonstrate the applicability of this polymerization technique to obtain a unique class of novel macromolecular structures with tailored properties.

## 2. Results and Discussion

**Scheme 2:**
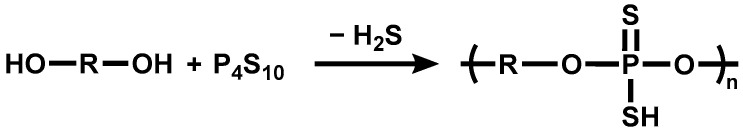
The formation of poly(dithiophosphate) in the reaction of a diol with phosphorus pentasulfide.

Preliminary experiments indicated that phosphorus pentasulfide (P_4_S_10_) successfully reacts with stoichiometric amounts of diols without the addition of any kind of catalyst, i.e., metals or metal compounds, in tetrahydrofuran (THF) at 60 °C, and this process results in poly(dithiophosphate)s (PDTPs), as shown in Scheme 2. Subsequently, a systematic series of experiments were designed to explore the reaction of P_4_S_10_ with various diols, such as poly(ethylene glycol) (PEG400), ethylene glycol (EG), 1,6-hexanediol (HD) and the mixtures of pairs of these diols by using stoichiometric amounts of the reactants, as presented in Table 1. In order to decrease the viscosity of the reaction medium and to provide a solvent for the reactants and product, THF was added to the reaction mixture. Visual observation indicated that P_4_S_10_ is insoluble in the mixture of the diols and THF, and it appears as a solid phase at the beginning of the reaction. However, this solid material disappears in the course of the reaction, indicating the formation of a compound soluble in the liquid phase. After the workup process (evaporation of the solvent and drying in vacuo, see Experimental), the dried products were obtained as colorless viscous liquids, with yields higher than 95% in all cases.

**Figure 1:**
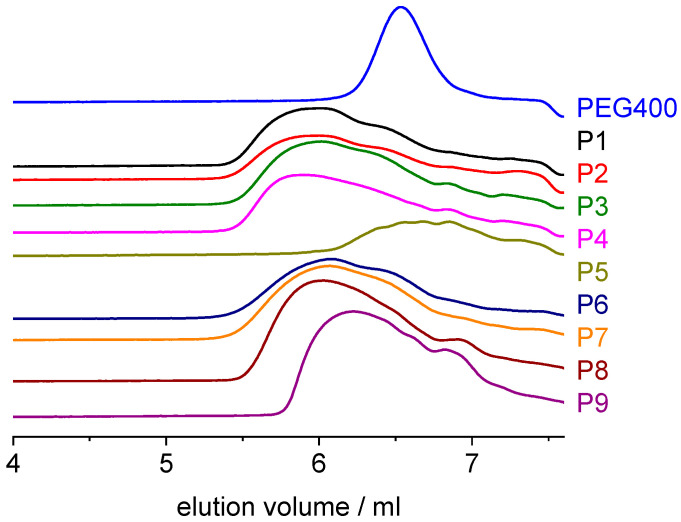
The gel permeation (GPC) chromatograms of PEG400 and P1–P9 PDTP samples.

**Figure 2:**
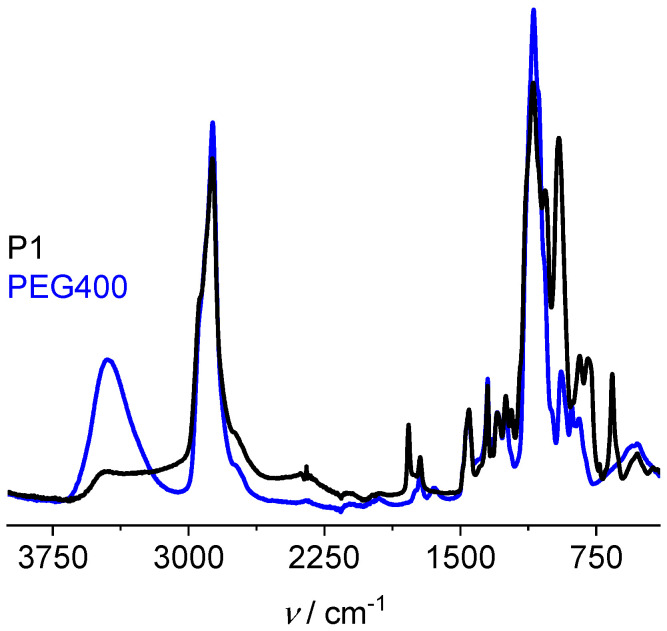
The FTIR spectra of PEG400 and the P1 PDTP sample.

First, the reaction of P_4_S_10_ was investigated with PEG400 as the diol (P1 sample in Table 1). Gel permeation chromatography (GPC) measurements showed that the main fraction of the product consisted of macromolecules with a higher hydrodynamic radius, i.e., higher molecular weight, than those of the PEG400 reagent (Figure 1). This indicates the coupling of PEG400 molecules. FTIR measurements were also performed on the obtained polymer P1 (Figure 2). A comparison of the IR absorption spectra of PEG400 and P1 implies that the intensity of the broad peak at 3450 cm^−1^, belonging to the O-H stretching in PEG400, decreased considerably in P1, indicating the reaction of the chain end –OH groups of PEG400 during the polymerization. The absorption peaks in the spectrum of P1 at 797 and 667 cm^−1^ are also in accordance with the structure forming in the reaction displayed in Scheme 2 because the latter peak belongs to the P=S stretching while the former can be assumed to be the band of the S-H in-plane scissoring [33].

**Figure 3:**
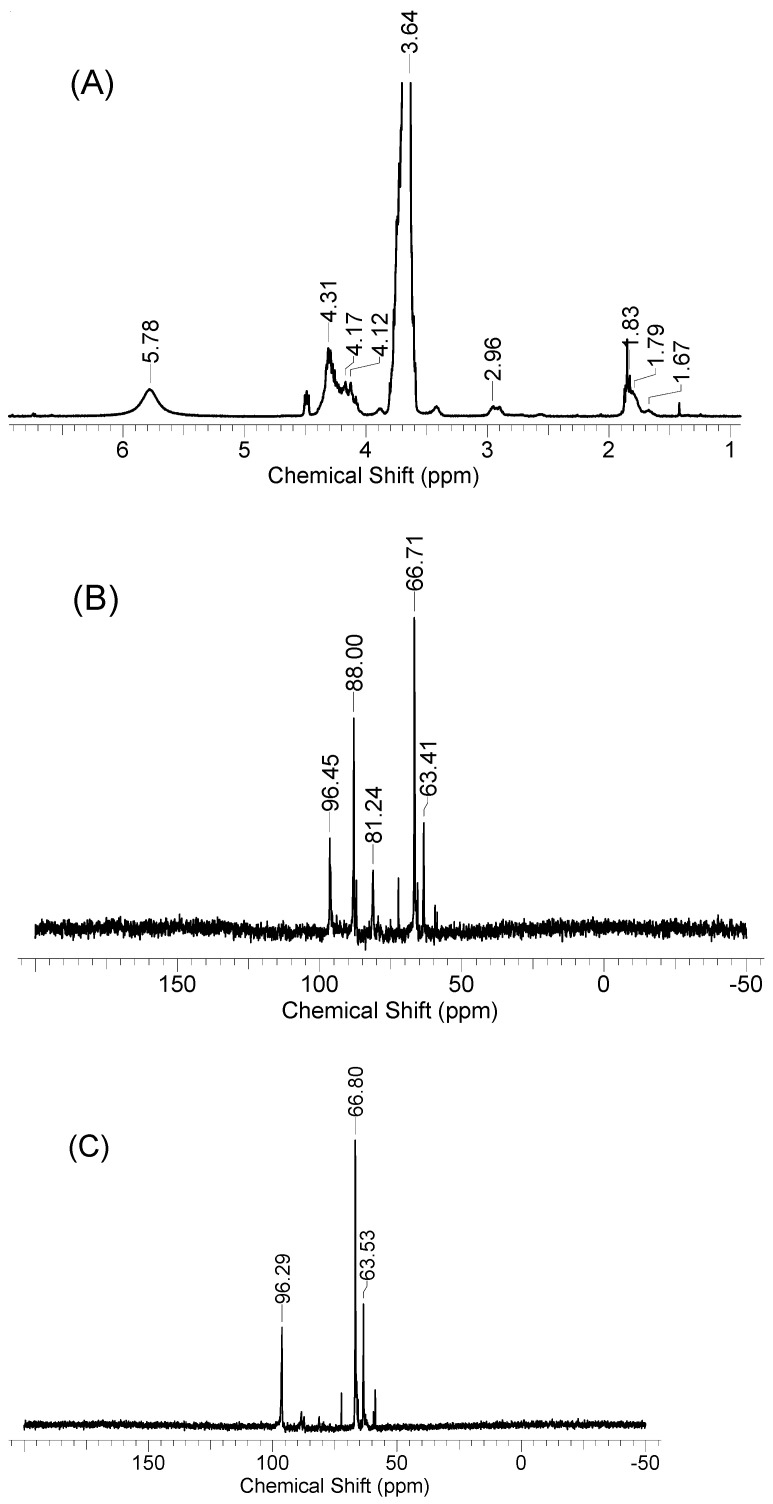
The ^1^H NMR (**A**) and the ^31^P NMR (**B**,**C**) spectra of the P1 PDTP sample recorded two days (**B**) and two and a half months (**C**) after the polymerization (solvent: CDCl_3_).

The chemical structure of the product was also investigated by ^1^H NMR spectroscopy (Figure 3A). The appearance of a broad signal at 5.78 ppm, belonging to the exchangeable acidic protons (and water impurity), and that at 4.3 ppm, assigned to the -CH_2_-O-P sequences, proves the occurrence of the reaction between PEG400 and P_4_S_10_.

**Scheme 3:**
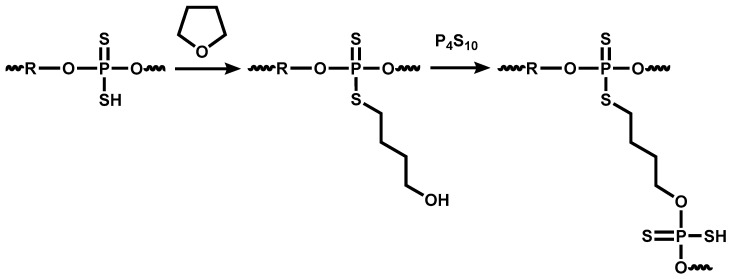
Branching point formation via the ring-opening reaction between the poly(dithiophosphate) and the tetrahydrofuran solvent molecule.

Interestingly, an unexpected side reaction occurs when using THF as a diluent during the polymerization process between P_4_S_10_ and diols. The ring-opening of THF molecules due to the nucleophilic attack of the P-SH group in the resulting polymers (Scheme 3, Figure S1) leads to the incorporation of such pendant molecular units in the polymer. The reaction between the poly(dithiophosphate) and THF is also proved by carrying out the polymerization process between P_4_S_10_ and PEG400 in toluene. In this case, the signals between 1.5 and 2.0 ppm in the ^1^H NMR spectrum of the sample are absent (Figure S2). Because new -CH_2_-OH groups appear in the reaction between THF and PDTP, this can lead to the formation of branching points, as depicted in Scheme 3. This is also indicated by the appearance of signals at 4.09 and 4.19 ppm in the ^1^H NMR spectrum (Figure 3A). From the integration of the peaks in the ^1^H NMR spectrum, it can be obtained that the ratio of the incorporated THF:PEG400 units is roughly 1:6. This unexpected finding indicates the unique opportunity of a broad range of post-polymerization modifications of poly(dithiophosphate)s by ring-opening reactions similar to the one observed with THF in this case.

**Scheme 4:**
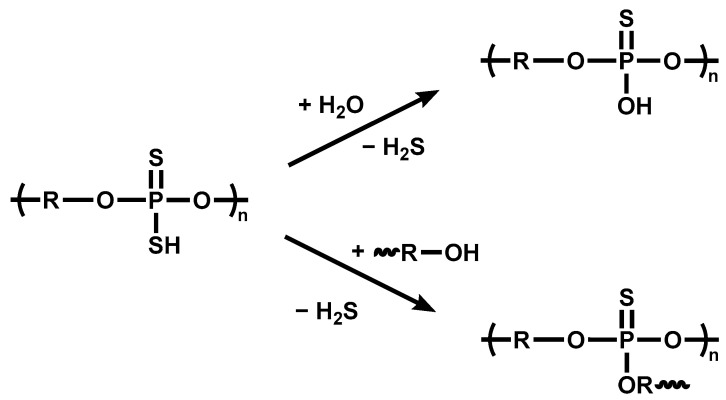
The hydrolytic and esterification reactions of the poly(dithiophosphate), the latter also leading to branching.

The presence of phosphorus in the polymer product was proved by ^31^P NMR spectroscopy. The desired product (Scheme 2) is formed in high conversion, as proved by the ^31^P NMR signal at 88 ppm [34]. However, additional coproduct formation is also observed. By comparing the ^31^P spectra recorded after 2 days (Figure 3B) and 2.5 months (Figure 3C) of polymerization, considerable differences can be observed. The signals at 88 and 81 ppm, assigned to the (RO)_2_P(S)SH units, disappeared during the 2.5 months. The signal at 96 ppm, which can be assigned to the (RO)_2_P(S)(SCH_2_-) structure, does not change during the storage, and the signals at 67 and 64 ppm are also present in both spectra. These two signals can be assigned to the phosphorus atom in (RO)_2_P(S)(OR) at 67 ppm and in (RO)_2_P(S)OH at 64 ppm. The absence of any signals around 0 ppm indicates that the product does not contain totally hydrolyzed phosphoric acid units; only thiolated phosphorus centers exist in these polymers. These observations can be explained by assuming two reactions, the hydrolysis reaction of the phosphorus-SH moiety with traces of water and the esterification with the unreacted terminal hydroxyl groups, resulting in the formation of additional branching points (Scheme 4). These assumptions are in accordance with previous investigations on the hydrolysis of zinc dialkyl dithiophosphates [35]. The occurrence of esterification is also confirmed by the determination of the ^1^H NMR signal areas of CH_2_OP and other CH_2_O protons, which increases with time, considering the spectra recorded after two days and two and a half months after the polymerization.

**Figure 4:**
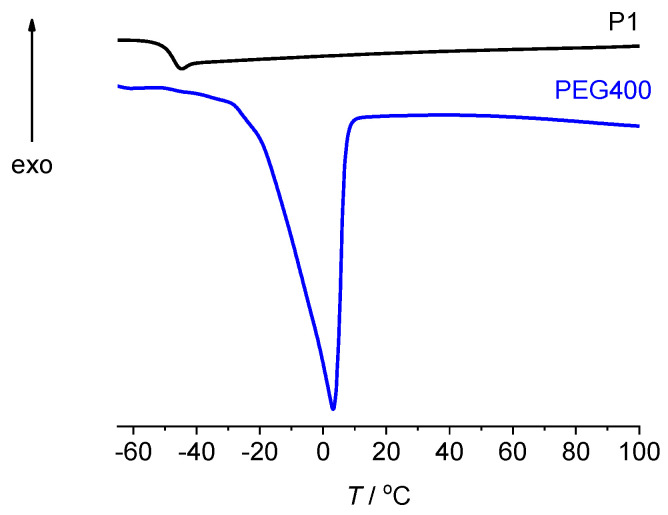
The differential scanning calorimetry (DSC) curves of PEG400 and the P1 PDTP sample.

**Figure 5:**
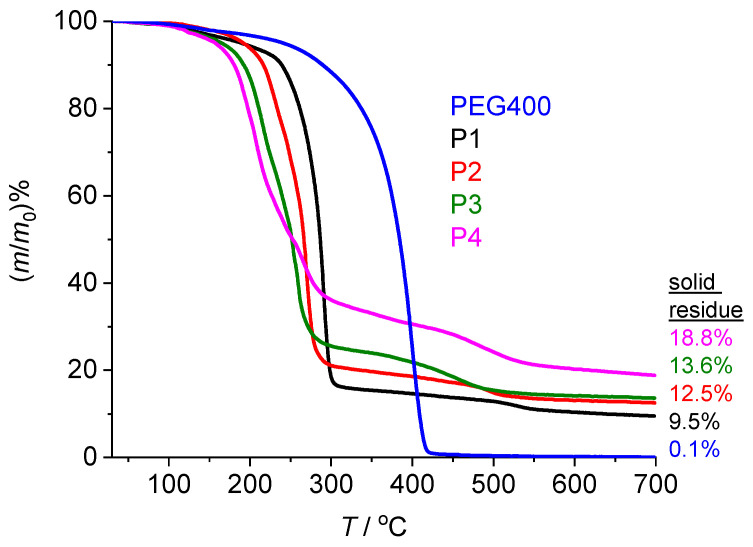
The thermogravimetric analysis (TGA) curves with the corresponding solid residue values of PEG400 and the P1–P4 PDTP samples.

Differential scanning calorimetric measurements show that while a melting peak appears at 2 °C on the curve of PEG400, indicating the crystallization of the PEG oligomers at low temperature, melting is absent on the curve of the P1 polymeric product and only a glass transition can be observed at −47 °C (Figure 4). This considerable difference in the thermal behavior indicates the hindrance of the appropriate orientation of the poly(ethylene glycol) segments required for crystallization due to the presence of the coupling (di)thiophosphate units. Thermogravimetric analysis (TGA) curves (Figure 5) also show the difference between P1 and PEG400 since P1 starts to degrade under an inert atmosphere at ca. 100 °C lower temperature than PEG400, indicating the thermal lability of the P-O-C bond. While the solid residue in the case of PEG400 is negligible at high temperatures, it is 9.5% in the case of the P1 poly(dithiophosphate) sample due to the formation of the resulting nonvolatile inorganic material.

The acidic character of P1 in aqueous conditions was also studied. It was titrated with NaOH solutions. The first step of the two-step titration curve indicates the presence of strong acidic groups, with p*K*_a_ = 3.5. This means that the P1 polymer behaves as a strong acidic polyelectrolyte in water (Figure S3).

Copolymerization experiments were performed with the simultaneous addition of PEG400 and ethylene glycol (Table 1). GPC measurements showed that there was no considerable difference between the hydrodynamic volume (and the molecular weight) of the formed copolymers P2–P4 (Figure 1). However, when only ethylene glycol was used as a diol (P5), a significantly lower hydrodynamic volume was observed for this product, indicating its lower molecular weight. From the comparison of the ^1^H NMR spectra of the P1–P4 samples, it can be concluded that the ratio of the integrals of the CH_2_-O-P signals (at 4.3 ppm) related to the CH_2_-O-C signals (at 3.5–3.9 ppm) increases with increasing EG content in the feed (Figure S4). This indicates that higher EG content in the feed results in lower P-P average distances in the polymer chains. This is indirect evidence of the ethylene glycol incorporation in the polymer chain. In the case of the copolymers P2–P4, the ^31^P NMR spectra also prove the partial hydrolysis and esterification side reactions (Figure S5). DSC measurements show slightly increasing *T*_g_ with increasing EG unit content in these copolymers. However, a considerable difference was only observed in the case of the high EG-containing copolymers, P4 and P5 (Table 1). Similarly, a slight decrease in the temperature of the first degradation step and an increase in the remaining solid residue ratio was observed in the TGA curves with increasing EG content (Figure 5). The latter observation is in accordance with the shortening of the average P-P distance, i.e., with the increment of the P-content in a given mass of polymers, with increasing EG content in the feed.

**Figure 6:**
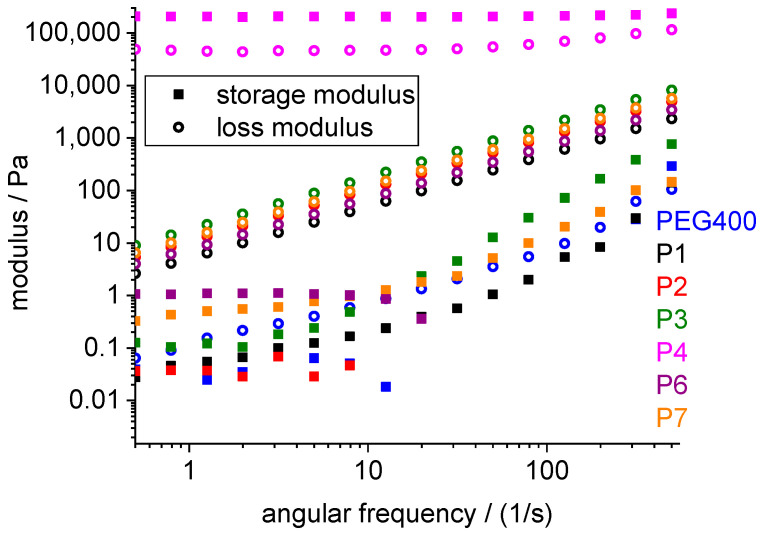
The storage (filled symbols) and loss (open symbols) moduli values of PEG400, P1–P4, P6 and P7 PDTP samples, obtained by oscillation rheometry.

The mechanical properties of the copolymers were determined by oscillatory rheological investigations. Based on the observed results shown in Figure 6, it can be concluded that the P1 sample has higher loss moduli than PEG400 due to its poly((di)thiophosphate) structure. The moduli data indicate that the mechanical properties of the obtained polymers can be varied within wide limits by changing the PEG400:EG ratio in the feed. The P1, P2 and P3 samples have nearly the same moduli values, and in accordance with their viscous liquid form, these materials possess considerably lower storage than loss moduli, while the storage modulus for the P2 sample cannot be detected over a wide frequency range, suggesting a completely liquid behavior (Figure 6). While the storage modulus shows frequency independence at low frequencies and nearly linear frequency dependence at high frequencies on a log–log scale, the loss modulus depends linearly on the frequency in the whole applied frequency range, with a slope of 1, typical for viscoelastic liquids. The fact that the storage modulus point series do not intersect with the loss moduli indicates that the relaxation times are shorter than the reciprocally applied high-frequency limit (2 ms), in accordance with the moderate molecular weights and branching structures of the obtained polymer chains. In the case of the physical network type copolymers P4 and P5, only the former could be measured by the applied oscillation rheometry setup. The P4 sample, which has high EG content, i.e., low average P-P distance, has much higher moduli values than the copolymers with higher PEG400 content. Moreover, in the case of the P4 sample, the storage and loss moduli are in the same order of magnitude and both are nearly frequency-independent, indicating the appearance of much stronger secondary intermacromolecular forces in this copolymer composition. The point series are similar to the “imperfect network” case with crosslinks (in this case, the H-bonds) and dangling chains [36].

**Figure 7:**
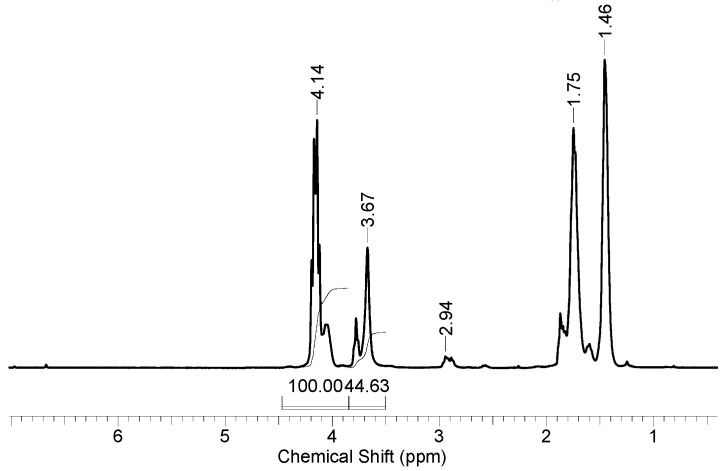
The ^1^H NMR spectrum of the P9 PDTP copolymer sample.

Copolymers were also synthesized with PEG400 and 1,6-hexanediol comonomers. As displayed in Figure 1, the peaks of the GPC curves of the P6, P7 and P8 copolymers have nearly the same elution volume as P1. Considerable molecular weight decrement can be observed in the case of P9 (Figure 1), similar to P5. Based on the ^31^P NMR spectra (Figure S6), the hydrolysis and esterification side reactions were also detected in the case of the polymers with 1,6-hexanediol monomeric units. The ^1^H NMR spectra show the signals of the -CH_2_- protons adjacent to the unreacted -OH groups (-CH_2_-OH) in the 3.4–3.85 ppm region and to the reacted ones (-CH_2_-O-P) between 3.9 and 4.5 ppm (Figure 7). In the case of P9, the conversion of the –OH groups (roughly 69%) can be determined by the comparison of the integrated area of these two signal groups. This relatively low conversion value means that there is a rather high number of unreacted –OH groups, which results in a reasonably high concentration of intermolecular H-bonds, explaining the observed physical network formation at room temperature. Similar physical network consistency can also be found in the case of the P8 copolymer. However, a higher increment in the PEG400 addition in the feed, similar to the EG-containing copolymers P2–P5, results in polymers as viscous liquids at room temperature. The oscillatory rheology response of these copolymers (P6 and P7) shows that the moduli are in the same region and the frequency response is similar to that of the P1–P3 samples. On the DSC curves, a glass transition can be observed at around −45 °C, independent of the composition (Table 1).

## 3. Materials and Methods

### 3.1. Materials

P_4_S_10_, poly(ethylene glycol) (*M*_n_ = 400 g/mol) (PEG400) and 1,6-hexanediol (HDO) were purchased from Merck (Sigma Aldrich, Darmstadt, Germany), while ethylene glycol (EG) was purchased from Molar Chemicals Ltd. (Halásztelek, Hungary) and tetrahydrofuran from VWR (Debrecen, Hungary). All the chemicals were used as received.

### 3.2. Polymer Syntheses

P_4_S_10_ (200–400 mg), a selected diol or mixture of diols and tetrahydrofuran were measured into a vial in stoichiometric amounts (i.e., 1 mole P_4_S_10_ to 4 mole diol). The molar ratio of the different diols was systematically varied (Table 1). The reaction mixtures were diluted with tetrahydrofuran to obtain 1 g/mL reactant/THF ratios and stirred at 60 °C for 6 h. Afterwards, the mixture was diluted with ca. 3 mL tetrahydrofuran and filtered using a 0.45 µm syringe filter, followed by the removal of the solvent under reduced pressure. The dried products were obtained after vacuum drying at 60 °C until constant weight. The reaction in toluene was carried out the same way.

### 3.3. Characterization Methods

^1^H and ^31^P NMR spectra were recorded on a Varian 300 MHz spectrometer at 30 °C in CDCl_3_.

The gel permeation chromatography (GPC) system was equipped with Waters Styragel HR1 and HR4 columns, a Waters 515 HPLC pump, a Waters 717 autosampler, a Jetstream column thermostat and an Agilent 1260 Infinity refractive index detector. Tetrahydrofuran was used as an eluent, with a 0.3 mL/min flow rate at 35 °C. Calibration was made with the polystyrene standards of narrow molecular weight distribution (from PSS Polymer Standards Services GmbH, Mainz, Germany).

Alkalimetric titration curves were recorded using a VWR MD8000L pH meter. For the titrations, 20–40 mg of the polymer was dissolved in 50 mL of distilled water. The titrant, a 0.013 M NaOH solution, was added by a syringe pump at a 10 mL/h rate.

Differential scanning calorimetry (DSC) measurements were performed on Mettler Toledo DSC821e equipment in the −80 to +100 °C temperature range, with a 10 °C/min heating rate in pierced 40 μL alumina crucibles under a nitrogen atmosphere with an 80 mL/min flow rate. The second heating curves were evaluated, and the inflection points of the glass transitions were regarded as glass transition temperatures.

TGA curves were recorded on a Setaram LabsysEvo (Lyon, France) TG-DSC system in a flowing (90 mL/min) nitrogen atmosphere in the 25 to 700 °C temperature range with a heating rate of 10 °C/min.

Oscillatory rheology measurements, applicable for determining the mechanical behavior of samples with a liquid consistency, were performed on an Anton Paar Physica MCR 301 rheometer at 25 °C with a cone-plate geometry probe (diameter: 25 mm; cone angle: 1°; sample gap: 0.054 mm) applying 1% strain in the 0.5–500 1/s angular frequency range.

## 4. Conclusions

An unexplored process, namely, the reaction between phosphorus pentasulfide (P_4_S_10_) and diols such as ethylene glycol (EG), 1,6-hexanediol (HD) and poly(ethylene glycol) (PEG), was investigated for the synthesis of a novel class of functional phosphorus- and sulfur-containing polymers, poly(dithiophosphate)s (PDTPs) (see Scheme 2), under mild conditions, that is, without any catalyst in the presence of THF or toluene diluents at a low temperature, i.e., at 60 °C, in contrast to the only reported case at 120–180 °C [30]. It was found that this simple process successfully results in PDTPs with higher than 95% yields. The structure of the resulting polymers was revealed by FTIR, ^1^H and ^31^P NMR spectroscopies and GPC measurements. Interestingly, the formation of branching points was detected in the case using THF as a diluent, which is attributed to the ring-opening of this molecule by the pendant sulfide group in the polymer chain. In contrast, this process is absent when toluene is used. By reacting P_4_S_10_ with mixtures of PEG and EG or HD, PDTP copolymers were obtained. The prepared PDTPs possess low glass transition temperatures (*T_g_*s) in the range of −4 to −50 °C, indicating the formation of elastic chains. It is important to note that there is no crystalline fraction of PEG in the PDTPs. This can be attributed to the prevention of the necessary orientation and packing of the PEG chains in PDTPs. It has to be noted that this behavior might be useful in such areas as ion-conducting membranes for batteries. The thermogravimetric analyses show that the investigated PDTPs have lower thermal stability than PEG, which is attributed to the lower stability of the P-O-C bonds than the C-O-C ether bonds in PEG. Testing the mechanical properties of the investigated PDTPs by oscillatory rheology measurements has indicated that the moduli of these polymers can be varied in a broad range, depending on the composition, i.e., on the ratio of the applied diol comonomers. In sum, it can be concluded that the reaction of P_4_S_10_ with diols under mild conditions enables the synthesis of a variety of PDTPs with interesting properties. These findings are expected to open new routes for polymer synthesis and various application possibilities, such as lithium or hydrogen ion-conducting polymer electrolytes for batteries and fuel cells, flame retardants, pH-responsive polymers, etc., with the involvement of the novel poly(dithiophosphate)s.

## Figures and Tables

**Table 1:** The diol ratios in the syntheses of poly(dithiophosphate)s, the glass transition temperature (*T*_g_) values and the peak molecular weights (*M_peak_*) of the resulting polymers (reaction conditions: 1 g/mL reactant/THF ratio, 60 °C, 6 h).

Sample	Diol Ratios in the Feed (Mol%)			*T*g (°C) *	*M_peak_* (g/mol) **
	PEG400	EG	HD		
P1	100	0	0	−47	1700
P2	75	25	0	−44	1700
P3	50	50	0	−42	1600
P4	25	75	0	−34	2200
P5	0	100	0	−4	400
P6	75	0	25	−47	1400
P7	50	0	50	−46	1500
P8	25	0	75	−43	1600
P9	0	0	100	−45	1000

* Determined by differential scanning calorimetry (DSC). ** Determined by gel permeation chromatography (GPC).

## Data Availability

Additional data on polymer analyses can be obtained by request from the corresponding authors.

## Supplementary Materials

The following supporting information can be downloaded at: https://www.mdpi.com/article/10.3390/ijms232415963/s1.

## Abbreviations

DSC	differential scanning calorimetry
DTP	dialkyl dithiophosphate
EG	ethylene glycol
FTIR	Fourier-transform infrared spectroscopy
GPC	gel permeation chromatography
HD	1,6-hexanediol
*M*_n_	number average molecular weight
*M_peak_*	peak molecular weight
NaOH	sodium hydroxide
NMR	nuclear magnetic resonance spectroscopy
P_4_S_10_	phosphorus pentasulfide
PDTP	poly(dithiophosphate)
PEG	poly(ethylene glycol)
PEG400	poly(ethylene glycol) with 400 g/mol number average molecular weight
*T*_g_	glass transition temperature
TGA	thermogravimetric analysis
THF	tetrahydrofuran
