# Supplementary material for: Poly(dithiophosphate)s, a New Class of Phosphorus- and Sulfur-Containing Functional Polymers by a Catalyst-Free Facile Reaction between Diols and Phosphorus Pentasulfide
Source: Int J Mol Sci. 2022 Dec 15;23(24):15963. doi: 10.3390/ijms232415963 (PMC9787700; doi:10.3390/ijms232415963)
Supplement: Supplementary file 1 [file ijms-23-15963-s001.zip › ijms-2074402-supplementary.pdf]

SUPPORTING INFORMATION

Poly(dithiophosphate)s, a New Class of Phosphorus and Sulfur Containing Functional Polymers by a Catalyst-Free Facile Reaction between Diols and Phosphorus Pentasulfide

Ákos Szabó[1]*, Györgyi Szarka[1], László Trif[2], Benjámin Gyarmati[3], Laura Bereczki[4,5], Béla Iván[1]*, Ervin Kovács[1]*

[1]Polymer Chemistry and Physics Research Group, Institute of Materials and Environmental Chemistry, Research Centre for Natural Sciences, H-1117 Budapest, Magyar tudósok krt. 2, Hungary; szabo.akos@ttk.hu (Á.Sz.); szarka.gyorgyi@ttk.hu (Gy.Sz.); ivan.bela@ttk.hu (B.I.); kovacs.ervin@ttk.hu (E.K.)

[2]Functional Nanoparticles Research Group, Institute of Materials and Environmental Chemistry, Research Centre for Natural Sciences, H-1117 Budapest, Magyar tudósok krt. 2, Hungary; trif.laszlo@ttk.hu (L.T.)

[3]Soft Matters Group, Department of Physical Chemistry and Materials Science, Faculty of Chemical Technology and Biotechnology, Budapest University of Technology and Economics, H-1111 Budapest, Műegyetem rkp. 3, Hungary; gyarmati.benjamin@vbk.bme.hu (B. Gy.)

[4]Plasma Chemistry Research Group, Institute of Materials and Environmental Chemistry, Research Centre for Natural Sciences, H-1117 Budapest, Magyar tudósok krt. 2, Hungary; nagyne.bereczki.laura@ttk.hu (L.B.)

[5]Chemical Crystallography Research Laboratory, Research Centre for Natural Sciences, H-1117 Budapest, Magyar tudósok krt. 2, Hungary; nagyne.bereczki.laura@ttk.hu (L.B.)

CONTENT

Figure S1. The 1D TOCSY selective excitation spectra of the P1 sample. The peaks of the excited protons are highlighted in the ^{1}H NMR spectrum (black line).

Figure S2. The ^{1}H NMR spectrum of the product obtained in the reaction of P_4S_{10} and PEG400 carried out in toluene.

Evaluation of the 1D TOCSY measurements

Figure S3. Alkalimetric titration curve of the P1 sample.

Figure S4. The ^{1}H NMR spectra of the P1-P4 samples.

Figure S5. The ^{31}P NMR spectrum of the P3 copolymer after six months storage.

Figure S6. The ^{31}P NMR spectrum of the P9 sample.

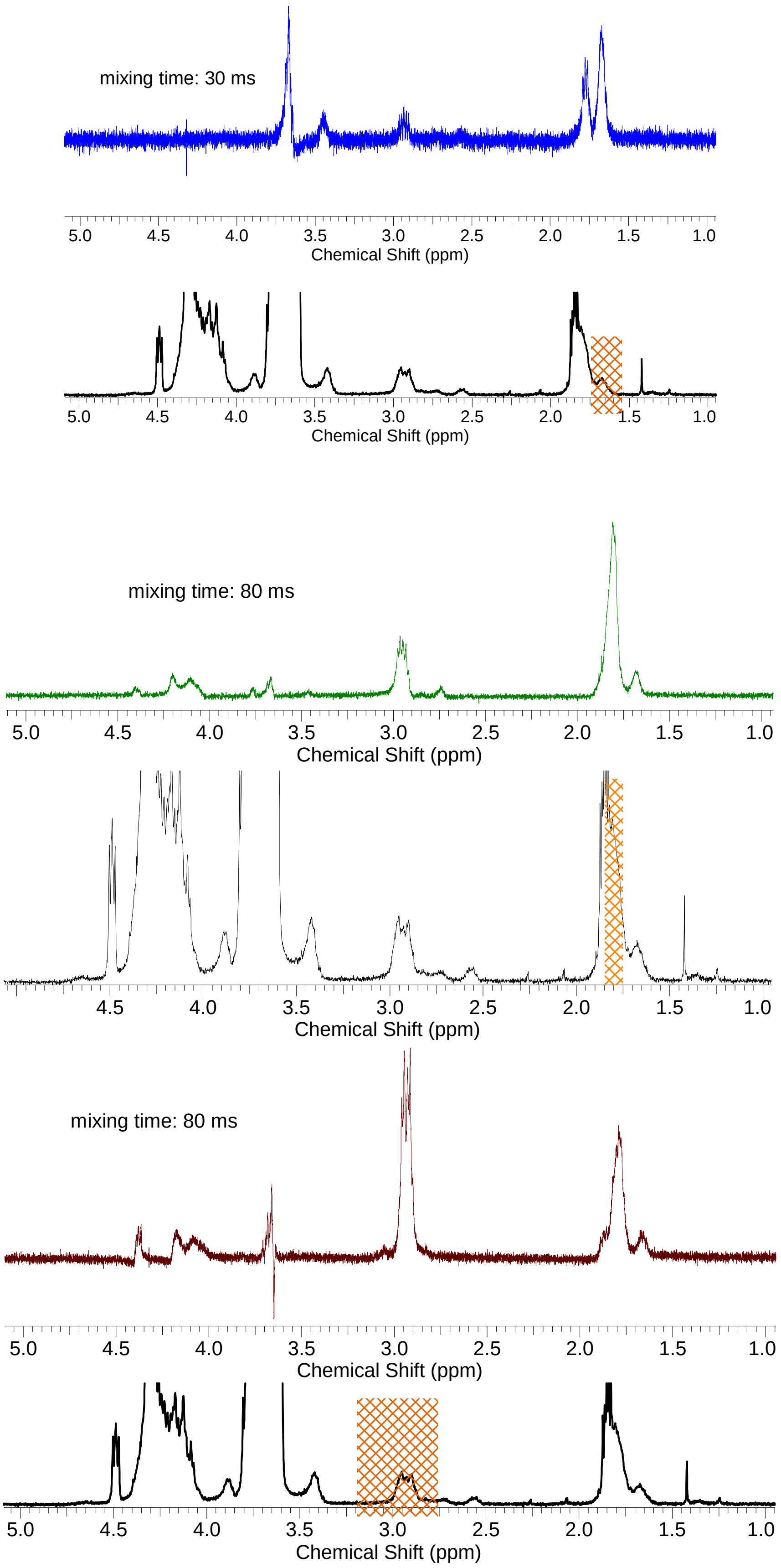

Figure S1. The 1D TOCSY selective excitation spectra of the P1 sample. The peaks of the excited protons are highlighted in the ^{1}H NMR spectrum (black line).

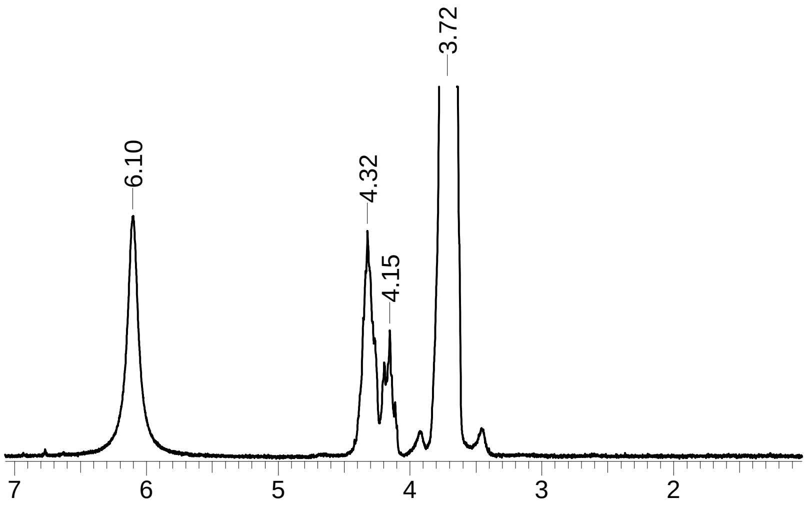

Figure S2. The ^{1}H NMR spectrum of the product obtained in the reaction of P_4S_{10} and PEG400 carried out in toluene.

Evaluation of the 1D TOCSY measurements

Exciting the ^{1}H nuclei giving the 1.67 ppm peak with short mixing time, it can be seen that the groups having the protons giving the signals at 3.67 ppm and 1.78 ppm are adjacent to the CH_2-type group. With lower intensity, i.e. coupled by more covalent bonds, they also correlate with the protons giving the 2.93 ppm and 3.44 ppm peaks. Exciting the 1.81 ppm peak protons with longer mixing time, the 4.09 ppm and 4.19 ppm peaks, are in the region of CH_2-O-P, and the peaks at 1.67 ppm and 2.93 ppm appear. The excitation of the 2.93 ppm peak protons, are typically S-CH_2, results in the appearance of all the above peaks. Considering these observations, the incorporation of THF molecules can be assumed (Scheme 3).

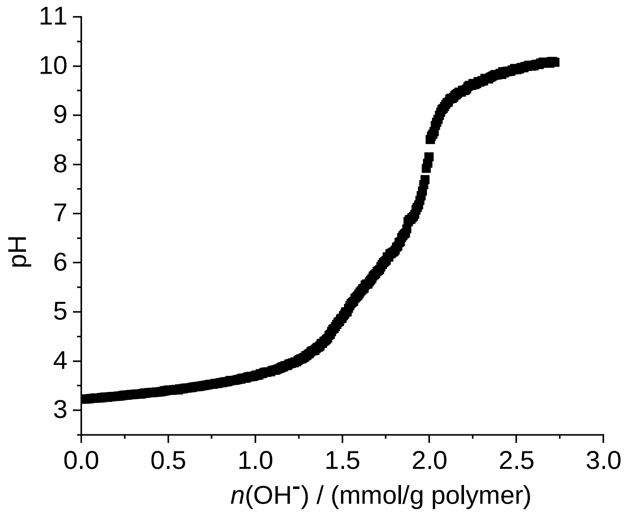

Figure S3. Alkalimetric titration curve of the P1 sample.

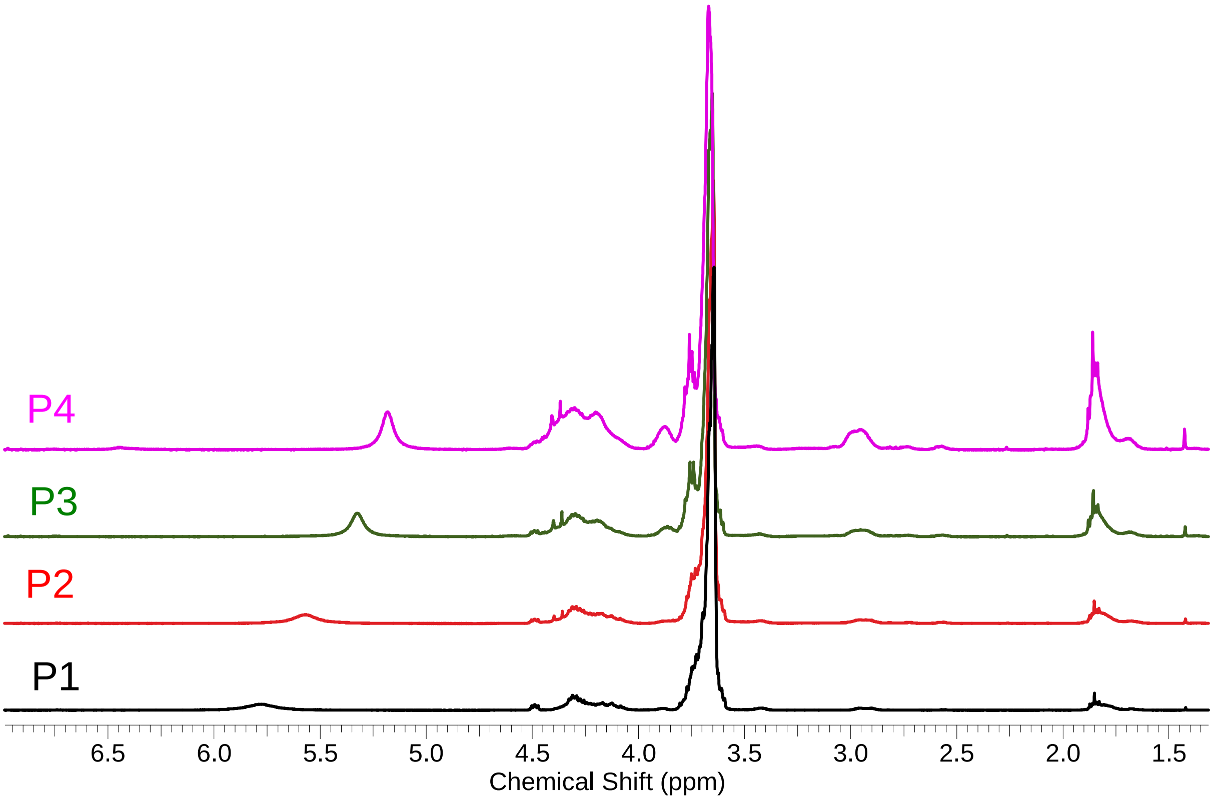

Figure S4. The ^{1}H NMR spectra of the P1-P4 samples.

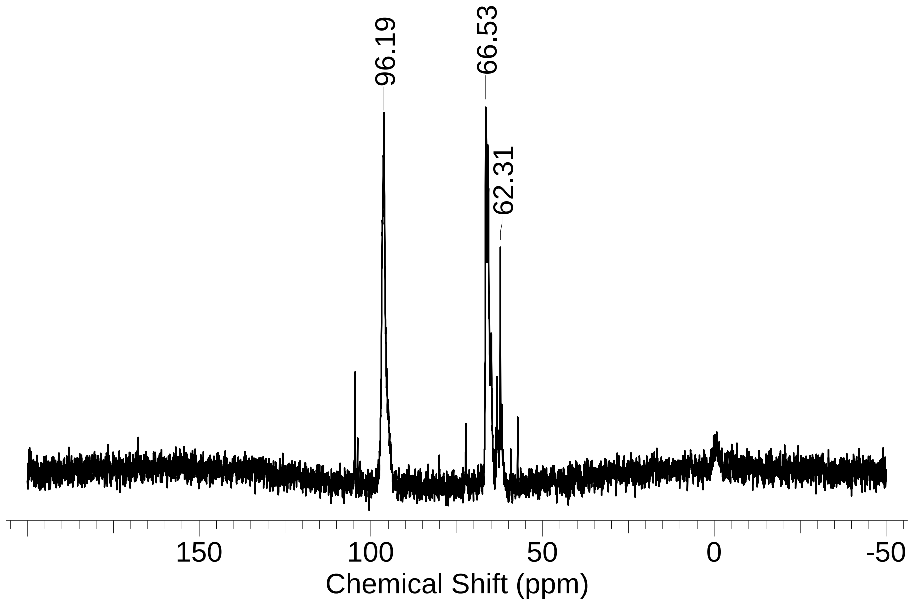

Figure S5. The ^{31}P NMR spectrum of the P3 copolymer after six months storage.

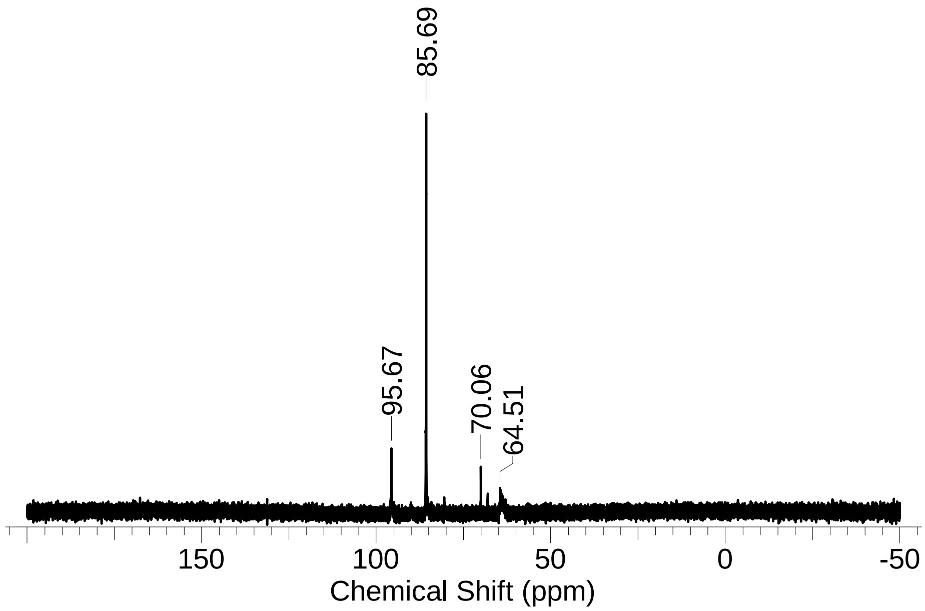

Figure S6. The ^{31}P NMR spectrum of the P9 sample.
